# Lipid Metabolism-Related Enzyme Inhibition and Antioxidant Potential of the Extracts and Phytochemical Compounds from *Trachystemon orientalis* (L.) D.Don

**DOI:** 10.1007/s11130-025-01358-z

**Published:** 2025-04-28

**Authors:** Tuğba Subaş, Merve Badem, Şeyda Kanbolat, Ufuk Özgen, Sıla Özlem Şener, Gül Yazıcı, Mevlüde İlknur Şeker

**Affiliations:** 1 Department of Pharmacognosy, Faculty of Pharmacy, Karadeniz Technical University, Trabzon, 61080 Turkey; 2 Department of Biochemistry, Faculty of Pharmacy, Karadeniz Technical University, Trabzon, Turkey; 3 Department of Pharmacognosy, Faculty of Pharmacy, University of Health Sciences, Ankara, Turkey

**Keywords:** Antioxidant, Cholesterol esterase, Danshensu, Pancreatic lipase, Rosmarinic acid, *β*-sitosterol

## Abstract

*Trachystemon orientalis* (L.) D.Don is a perennial herb extensively utilized for culinary purposes and the treatment of numerous ailments. Hyperlipidemia is a significant risk factor for atherosclerosis, diabetes, obesity, and coronary heart disease. The inhibition of pancreatic lipase (PL) and cholesterol esterase (CE) aims to mitigate problems associated with lipid metabolism disorders by decreasing hyperlipidemia. In this context, enhancing antioxidant capability is an additional objective. This study assessed the PL and CE inhibitory actions of extracts and compounds from the aerial parts of *T. orientalis*, utilizing ferric reducing antioxidant power (FRAP) and cupric-reducing antioxidant capacity (CUPRAC) assays to measure antioxidant capabilities. Rosmarinic acid, a combination of rosmarinic acid and danshensu (phenolic acids), and *β*-sitosterol (phytosterol) were isolated. *β*-sitosterol (IC_50_: 41.698 ± 1.982 µg/mL, 14.249 ± 1.209 µg/mL, respectively) followed by rosmarinic acid (IC_50_: 48.213 ± 2.490 µg/mL, 21.941 ± 3.785 µg/mL, respectively) demonstrated significant inhibitory effects on PL and CE. Additionally, rosmarinic acid (FRAP: 1274.400 ± 1.633 µM TEAC, CUPRAC: 1801.389 ± 5.500 µM TEAC) and its combination with danshensu (FRAP: 1405.067 ± 4.491, CUPRAC: 2174.167 ± 9.647 µM TEAC) exhibited high antioxidant activity. The ethyl acetate subextract (TOE) from which rosmarinic acid was isolated showed considerable enzyme inhibitory (IC_50_: 47.577 ± 0.931 µg/mL, 12.443 ± 1.233 µg/mL, for PL and CE inhibition, respectively) and antioxidant activity (FRAP: 1770.021 ± 4.583, CUPRAC: 2189.167 ± 5.401 µM TEAC). *T. orientalis* has been identified as a promising natural resource for the management of lipid metabolism-related disorders and for enhancing antioxidant activity.

**Supplementary Information:**

The online version contains supplementary material available at 10.1007/s11130-025-01358-z.

## Clinical Trial

Not applicable.

**Supplementary Information:**

The online version contains supplementary material available at 10.1007/s11130-025-01358-z.

## Introduction

Hyperlipidemia is a metabolic condition characterized by elevated triglyceride and cholesterol levels in the bloodstream, resulting from high-fat diets and poor lifestyles. Hyperlipidemia is a significant risk factor for illnesses associated with lipid metabolism, including atherosclerosis, coronary heart disease, diabetes, and obesity [1]. The prevalence of metabolic syndrome, encompassing conditions such as hypertension, obesity, insulin resistance, and dyslipidemia, ranges from 20 to 45%. By 2030, it is projected that 89% of men and 85% of women globally would be overweight or obese [2].

Pancreatic lipase (PL) is the principal lipase enzyme that facilitates the transformation of triglycerides into free fatty acids and glycerol, accounting for roughly 70% of digestion and absorption [3]. PL is essential in converting dietary triglycerides into absorbable lipids and is an attractive target for its ability to diminish fat absorption and decelerate lipid metabolism, therefore averting the buildup of body fat. Cholesterol esterase (CE) is found in bile, hydrolyzing cholesterol esters and aiding the transfer of free cholesterol from micelles to enterocytes. Besides cholesterol esters, it also hydrolyzes substrates including fat-soluble vitamins, phospholipids, and triglycerides. The inhibition of phospholipase (PL) and cholesterol esterase (CE) to prevent the absorption of triglycerides and cholesterol esters is a therapy strategy for hyperlipidemia and other lipid metabolism disorders [4]. The PL inhibitor orlistat is utilized for obesity management, whereas simvastatin is employed for hypercholesterolemia; nonetheless, both possess significant adverse effects [5]. Consequently, natural products serve as significant sources in the quest for safe and efficacious antihyperlipidemic medicines.

*Trachystemon orientalis* (L.) D.Don, referred to in Turkey as “kaldırayak, kaldirik, balıkotu, ıspıt, hodan, tamara, acı hodan,” is the sole species under the genus *Trachystemon* G. Don (Boraginaceae) [6]. It proliferates in the Black Sea Region, Eastern Bulgaria, and the Western Caucasus globally. It is a perennial herb that attains a height of 30–40 cm, with blue-purple blooms, large leaves, and a rhizome [7]. It is extensively utilized, particularly in the Black Sea area of Turkey, where its leaves, petioles, and flowers are ingested as vegetables. The leaves serve as a diuretic, expectorant, and for digestive issues; the whole plant is utilized for irritated wounds [8, 9]. Studies have shown that its phytochemical composition encompasses flavonoids, phenolic compounds, essential oils, fatty acids, tannins, resin, mucilage, saponins, choline, minerals and vitamins [7, 10–12]. Literature indicates that the plant demonstrates antioxidant [7, 12, 13], antimicrobial [7, 8], antifungal, herbicidal [6], antimutagenic; α-amylase and α-glucosidase [7], butyrylcholinesterase [13], tyrosinase, collagenase and, elastase enzyme inhibition, as well as photoprotective activities [14].

Research on the phytochemical and biological activities of the plant is restricted. The plant, traditionally used for intestinal disorders, has demonstrated antioxidant and inhibitory effects on digestive enzymes like α-amylase and α-glucosidase [7, 8]. In addition, rosmarinic acid, danshensu, globoidnan B, and rabdosiin, exhibiting PL inhibitory activity, were isolated from the underground parts [15]. The results indicate that the aerial parts of the plant may be abundant in phytochemicals exhibiting antihyperlipidemic activity via the inhibition of PL and CE. This study aims to assess the PL and CE inhibitory activities as well as the antioxidant capacity of the extracts and major compounds from the aerial parts of *T. orientalis*.

## Materials and Methods

The Materials and Methods section is presented as Supplementary material.

## Results and Discussion

### Isolation and Elucidation of the Compounds

**Fig. 1:**
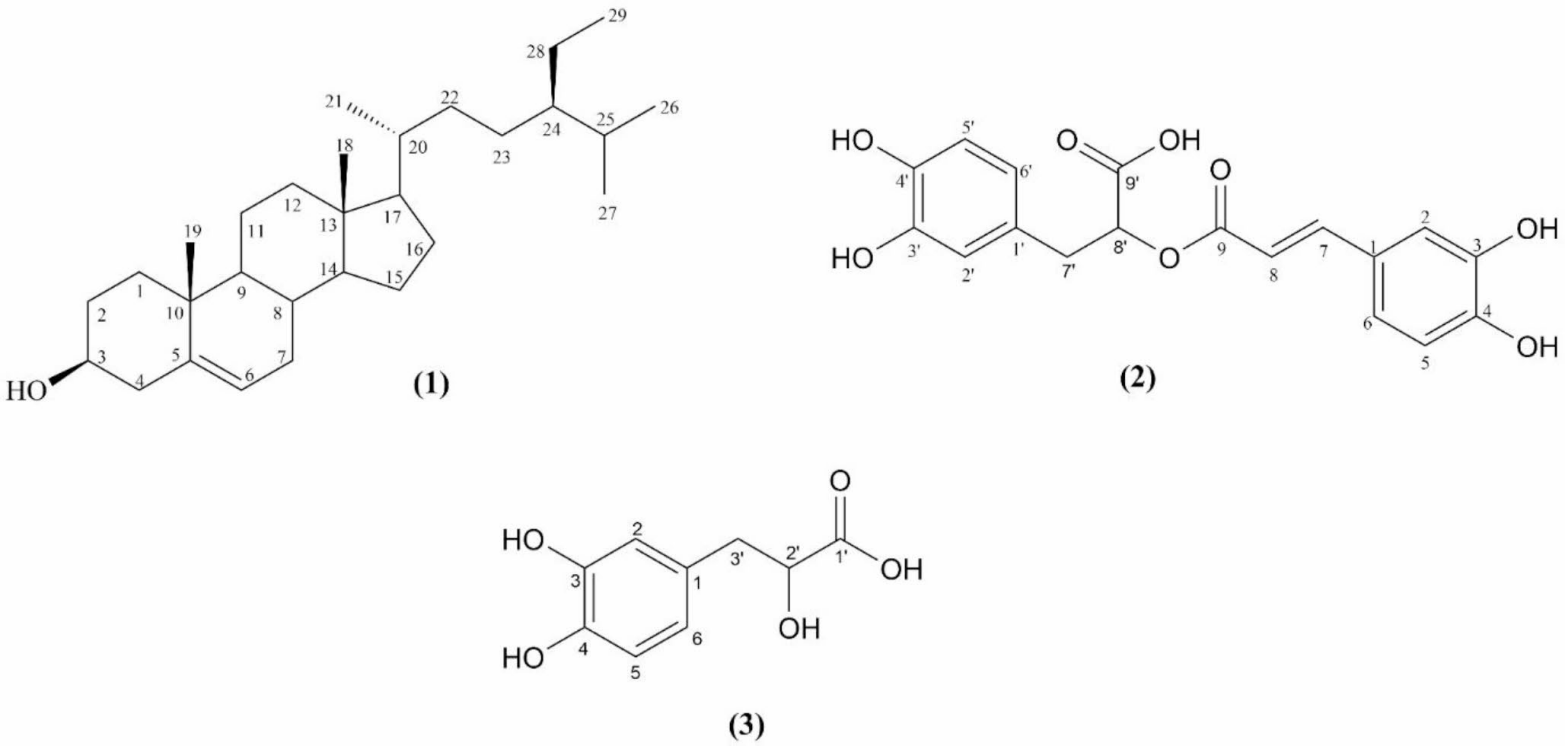
Structures of the compounds. (**1**) *β*-sitosterol, (**2**) Rosmarinic acid, (**3**) Danshensu

In this research, isolation studies were carried out on the subextracts prepared from the methanol extract (TOM), and the biological activities of the extracts and compounds were determined. The isolation studies resulted in the purification of three identified compounds (two phenolic acids and one sterol) by different chromatographic techniques (Figure S1). The structures of the isolated compounds were determined using 1D and 2D nuclear magnetic resonance (NMR) methods, with the spectra are presented in Figures S2-S12. *β*-sitosterol (**1**) from the chloroform subextract (TOC), rosmarinic acid (**2**) from the ethyl acetate subextract (TOE), and the mixture of rosmarinic acid and danshensu (**2** + **3**) from the remaining aqueous subextract (TOA) have been purified for the first time of the aerial parts of the plant (Fig. 1). The presence of *β*-sitosterol and rosmarinic acid in the plant has been demonstrated using HPLC method [12, 16]. Rosmarinic acid and danshensu were previously isolated from the underground parts of the plant [15].

### PL Inhibitory Activities of the Extracts and Compounds

**Fig. 2:**
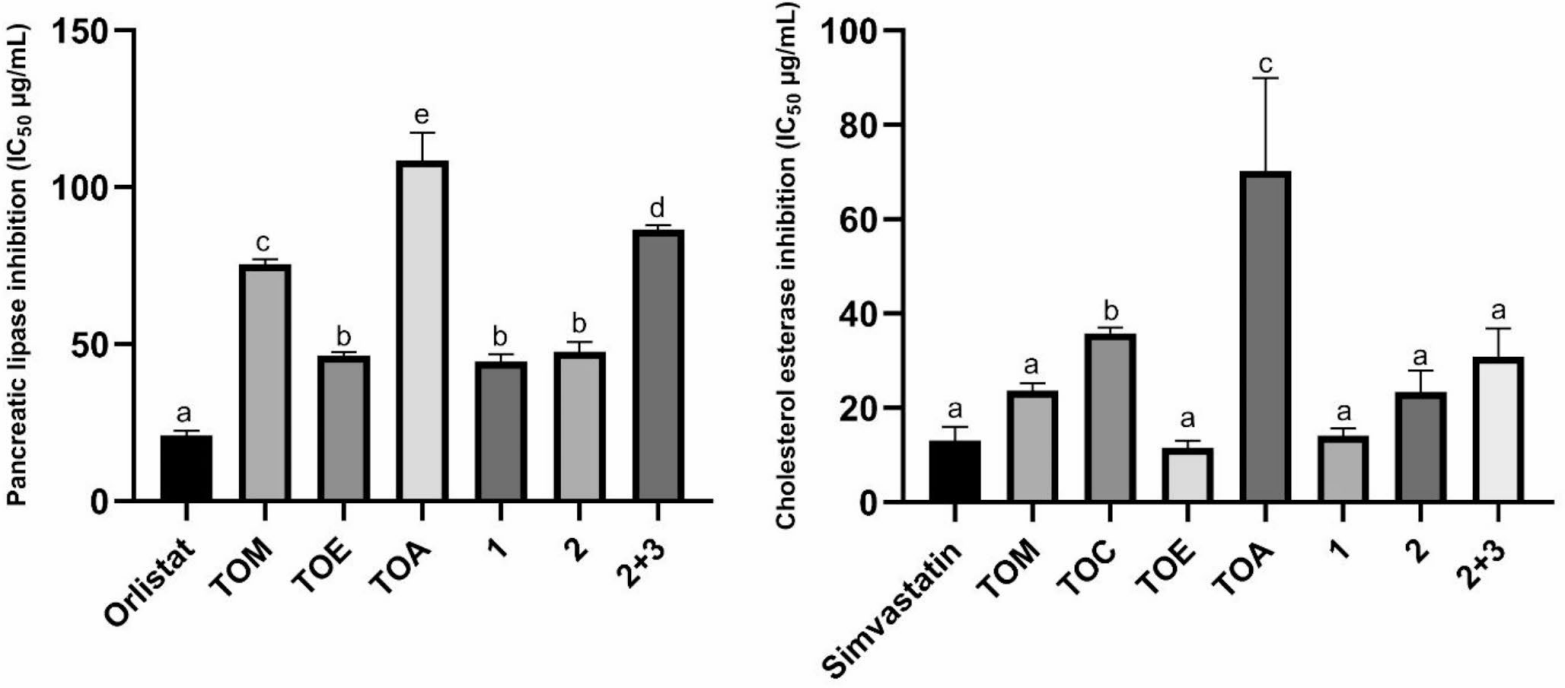
PL and CE inhibitory activity of the extracts and compounds. The values are represented by mean ± SD of triplicate measurements. Orlistat and simvastatin were used as positive control. Different letters (a > b > c > d) in the same graphic indicate significant differences (*p* < 0.05) by Tukey’s multiple comparisons test. (1) *β*-sitosterol (2) Rosmarinic acid, (3) Danshensu. TOM: The methanol extract of *Trachystemon orientalis*, TOC: The chloroform subextract of *T. orientalis*, TOE: The ethyl acetate subextract of *T. orientalis*, TOA: The remaining aqueous subextract of *T. orientalis*

The IC_50_ values for the extracts and the standard regarding PL inhibition are displayed in Table 1; Fig. 2. The efficacy of the extracts and compounds was weaker than orlistat (*p* < 0.0001). The activity of TOE surpassed that of the other extracts (IC_50_ = 47.577 ± 0.931 µg/mL, *p* < 0.0001). Among the isolated compounds, *β*-sitosterol (IC_50_ = 41.698 ± 1.982 µg/mL, *p* < 0.0001) and rosmarinic acid (IC_50_ = 48.213 ± 2.490 µg/mL, *p* < 0.0001) exhibited substantially greater efficacy than the combination of rosmarinic acid and danshensu. Rosmarinic acid, an ester of caffeic acid and 3,4-dihydroxyphenyl lactic acid (danshensu), is a significant phenolic acid [17]. Rosmarinic acid, the primary component of TOE, shown comparable activity to TOE, which may be ascribed to it. A research evaluated the PL inhibitory activity of *Rosmarinus officinalis* L. extract and pure rosmarinic acid, revealing IC_50_ values of 13.8 and 125.2 µg/mL, respectively. It has been suggested that rosmarinic acid and other phenolic compounds may have functioned synergistically [18]. In further investigations, rosmarinic acid shown significant lipase inhibitory activity (IC_50_ = 62.8 ± 2.7 µM, 49.421 ± 1.448 µg/mL), corroborating our findings [15, 19]. Danshensu (salvianic acid A) is a phenolic acid extracted from the roots of *Salvia miltiorrhiza* Bunge and is present in several Boraginaceae species [15, 20]. Danshensu has demonstrated the ability to suppress PL with an IC_50_ of 65.160 ± 4.443 µg/mL [15]. This study demonstrated that the combination of rosmarinic acid and danshensu had lower activity than rosmarinic acid alone. It may be inferred that rosmarinic acid and danshensu may exhibit antagonistic effects. The non-competitive binding of these two chemicals to the enzyme’s active site, or their simultaneous binding to distinct locations, may diminish their respective inhibitory effects. Furthermore, chemical or steric interactions among the compounds may further reduce their binding affinity to the enzyme.

This study demonstrated that *β*-sitosterol exhibits significant PL inhibitory activity. Literature has studies investigating the impact of *β*-sitosterol, a natural sterol, on PL, yielding contradictory results. In one study, similar to our findings (at 100 µg/mL, 68.47% inhibition), *β*-sitosterol (100 µg/mL) was found to inhibit PL by 79.1 ± 11.3% [21]. In another study, the compound (4 mg/mL) demonstrated considerable inhibition (IC_50_ = 82.56 mg/mL, 60.67% ± 0.53%) [22]. Other studies indicated that it exhibited little activity (2.99 ± 0.80%, 8.80 ± 4.15% inhibition) on PL [23, 24]. The divergent outcomes regarding the PL inhibitory action of *β*-sitosterol may stem from variations in experimental methodology, solvents, concentration, and the purity of the molecule. It was observed that *β*-sitosterol efficiently prevented adipogenesis in 3T3-L1 adipocytes, evidenced by a considerable reduction in intracellular triglyceride content and lipid accumulation, without including notable cytotoxicity [25].

### CE Inhibitory Activities of the Extracts and Compounds

All extracts and compounds demonstrated CE inhibitory activity (Table 1; Fig. 2). TOE had greater activity than simvastatin, however without statistical significance (IC_50_ = 12.443 ± 1.233 µg/mL). TOM, TOE (*p* < 0.0001), and TOC (*p* = 0.001) shown superior effect compared to TOA. *β*-sitosterol (IC_50_ = 14.249 ± 1.209 µg/mL) exhibited significant action comparable to that of simvastatin. This study is the first publication examining the CE inhibitory action of rosmarinic acid and *β*-sitosterol.

Studies demonstrates the impact of isolated compounds on hyperlipidemia via several pathways. While direct evidence of *β*-sitosterol, rosmarinic acid, or danshensu suppressing CE is lacking, their influence on lipid metabolism implies a possible function in regulating cholesterol homeostasis. *β*-sitosterol was observed to decrease cholesterol absorption and plasma cholesterol levels in hypercholesterolemic hamsters more effectively than sesamin. It was also discovered to diminish the transfer of cholesterol into micelles, maybe due to its structural similarity to cholesterol [26]. Rosmarinic acid (100 mg/kg) was found to improve cholesterol and triglyceride levels comparably to simvastatin in hypercholesterolemic rats on a high-fat diet [27]. Rosmarinic acid may be effective in combating hyperlipidemia through the regulation of lipid metabolism, antioxidant indicators, and inflammatory pathways [28]. Danshensu was found to reduce lipid accumulation in RAW264.7 macrophage cells by increasing intracellular cholesterol efflux [29].

**Table 1:** PL and CE inhibitory activities of the extracts and compounds

Extracts/Compounds	Enzyme inhibitory activities IC_50_ (µg/mL) ± SD^**^	
	Pancreatic lipase (PL)	Cholesterol esterase (CE)
TOM	77.255 ± 1.237^c^	23.572 ± 1.283^a^
TOC	nd^***^	37.379 ± 0.966^b^
TOE	47.577 ± 0.931^b^	12.443 ± 1.233^a^
TOA	114.035 ± 7.256^e^	67.945 ± 16.177^c^
*β*-sitosterol	41.698 ± 1.982^b^	14.249 ± 1.209^a^
Rosmarinic acid	48.213 ± 2.490^b^	21.941 ± 3.785^a^
Rosmarinic acid + Danshensu	88.003 ± 1.140^d^	28.491 ± 5.011^a^
Positive control^*^	22.709 ± 1.299^a^	13.981 ± 2.367^a^

^*^Siµvastatin for CE; Orlistat for PL. ^**^: Standard deviation, ^***^: not determined. Different letters (a > b > c > d) in the same column indicate significant differences (*p* < 0.05) by Tukey’s multiple comparisons test. TOM: The methanol extract of *Trachystemon on orientalis*, TOC: The chloroform subextract of *T. orientalis*, TOE: The ethyl acetate subextract of *T. orientalis*, TOA: The remaining aqueous subextract of *T. orientalis*

## Antioxidant Activity Results

The antioxidant capacities of the extracts and compounds, as assessed by the FRAP and CUPRAC assays, are presented in Table 2. The calibration curves for FRAP and CUPRAC assays are presented in Figure S13. TOE has the highest activity as indicated by both assays (FRAP = 1770.021 ± 4.583, CUPRAC = 2189.167 ± 5.401 µM TEAC). The combination of rosmarinic acid and danshensu exhibited superior antioxidant activity compared to rosmarinic acid alone (FRAP = 1405.067 ± 4.491, CUPRAC = 2174.167 ± 9.647 µM TEAC).

Oxidative stress is a significant risk factor associated with metabolic disorders, including hyperlipidemia, and may also influence enzyme activity [30]. Ethyl acetate extracts often include a high concentration of polyphenols, including rosmarinic acid. Consequently, they significantly impede free radical processes, mostly owing to their redox characteristics. Biyik et al. [16] asserted that the elevated antioxidant activity of *T. orientalis* is associated with rosmarinic acid. The combination of rosmarinic acid with danshensu, a significantly more polar molecule, has enhanced antioxidant potential. A research assessing the antioxidant activity of *S. miltiorrhiza* extracts identified danshensu as the most important marker for antioxidant effect [31]. Another investigation indicated that rosmarinic acid and its metabolite danshensu had radical scavenging ability equivalent to that of quercetin [32]. The TOA from which this mixture was obtained seems to have diminished antioxidant ability. The impact is likely diminished by the presence of other minor compounds in the extract. *β*-sitosterol and the TOC from which it was isolated had low antioxidant capacity. Ertas et al. [33] determined the antioxidant activity of *β*-sitosterol to be A_0.5_: 22.17 ± 0.98 µg/mL using the CUPRAC assay. *β*-sitosterol functions as a mild to moderate antioxidant and has demonstrated protective benefits against oxidative damage in in vivo experiments [34].

**Table 2:** FRAP and CUPRAC values of the extracts and compounds

Extracts/Compounds	FRAP^*^	CUPRAC^**^
TOM	500.147 ± 2.033	489.861 ± 2.508
TOC	nd^***^	86.528 ± 2.051
TOE	1770.021 ± 4.583	2189.167 ± 5.401
TOA	540.132 ± 7.937	107.639 ± 3.987
*β*-sitosterol	212.067 ± 5.312	91.111 ± 2.265
Rosmarinic acid	1274.400 ± 1.633	1801.389 ± 5.500
Rosmarinic acid + Danshensu	1405.067 ± 4.491	2174.167 ± 9.647

^*^FRAP value indicates iron reducing antioxidant power (µM Trolox equivalent/gram). ^**^CUPRAC value indicates copper reducing antioxidant power (µM Trolox equivalent/gram), ^***^: not determined. TOM: The methanol extract of *Trachystemon on orientalis*, TOC: The chloroform subextract of *T. orientalis*, TOE: The ethyl acetate subextract of *T. orientalis*, TOA: The remaining aqueous subextract of *T. orientalis*

## Conclusions

This study demonstrated that extracts and isolated compounds from *T. orientalis* shown inhibitory effects on PL and CE, as well as potential in regulating lipid metabolism. Among the tested extracts, TOE, and pure compounds, *β*-sitosterol and rosmarinic acid exhibited strong enzyme inhibition, suggesting their potential therapeutic application in lipid metabolism disorders. Our findings indicate that *T. orientalis* possesses considerable promise as a natural resource for the management of hyperlipidemia, principally due to its inhibition of lipid metabolism-related enzymes and its antioxidant capabilities. However, more extensive investigations are necessary to fully clarify its therapeutic potential, and the results of this study offer essential data for future research. Future research should concentrate on the impact of *T. orientalis* and its bioactive components on distinct lipid metabolism pathways to clarify its precise mechanism of action. Furthermore, in vivo investigations are necessary to evaluate its pharmacokinetic characteristics, bioavailability, and systemic effects. *T. orientalis* has potential for creating innovative nutraceutical and pharmaceutical products for hyperlipidemia and other lipid metabolism disorders.

## Electronic Supplementary Material

Below is the link to the electronic supplementary material.

Supplementary Material 1

## Data Availability

No datasets were generated or analysed during the current study.
