# Supplementary material for: Lipid Metabolism-Related Enzyme Inhibition and Antioxidant Potential of the Extracts and Phytochemical Compounds from Trachystemon orientalis (L.) D.Don
Source: Plant Foods Hum Nutr. 2025 Apr 28;80(2):114. doi: 10.1007/s11130-025-01358-z (PMC12037426; doi:10.1007/s11130-025-01358-z)
Supplement: Supplementary file 1 — Supplementary Material 1 [file 11130_2025_1358_MOESM1_ESM.docx]

**Supplementary Material**

**Lipid Metabolism-Related Enzyme Inhibition and Antioxidant Potential of the Extracts and Phytochemical Compounds from *Trachystemon orientalis* (L.) D.Don**

Tuğba Subaş^1^, Merve Badem^2^, Şeyda Kanbolat^2^, Ufuk Özgen^1^, Sıla Özlem Şener^3^, Gül Yazıcı^1^, Mevlüde İlknur Şeker^1^

^1^ Department of Pharmacognosy, Faculty of Pharmacy, Karadeniz Technical University, Trabzon, Turkey [tugbasubas@ktu.edu.tr](mailto:tugbasubas@ktu.edu.tr), 0000-0002-0956-6567; [uozgen@ktu.edu.tr](mailto:uozgen@ktu.edu.tr), 0000-0001-9839-6717; [yzcgul2503@gmail.com](mailto:yzcgul2503@gmail.com); [ilknurseker7@gmail.com](mailto:ilknurseker7@gmail.com)

^2^ Department of Biochemistry, Faculty of Pharmacy, Karadeniz Technical University, Trabzon, Turkey [mervecolakoglu@ktu.edu.tr](mailto:mervecolakoglu@ktu.edu.tr), 0000-0002-1265-5616; [seydaakkaya@ktu.edu.tr](mailto:seydaakkaya@ktu.edu.tr), 0000-0001-7261-7067

^3^ Department of Pharmacognosy, Faculty of Pharmacy, University of Health Sciences, Ankara, Turkey [silaozlem.sener@sbu.edu.tr](mailto:silaozlem.sener@sbu.edu.tr), 0000-0001-7679-7165

Corresponding Author: Tuğba SUBAŞ

E-mail adress: [tugbasubas@ktu.edu.tr](mailto:tugbasubas@ktu.edu.tr)

Department of Pharmacognosy, Faculty of Pharmacy, Karadeniz Technical University, Trabzon 61080, Turkey, 04623778914 – +905392009087

**Material and Methods**

**Chemicals and Instrumentation**

Ethyl acetate, chloroform, methanol, *n*-hexane, ethanol, 6-hydroxy-2,5,7,8-tetramethylchroman-2-carboxylic acid (Trolox), 2,4,6-tripyridyl-s-triazine (TPTZ), vanillin, *p*-nitrophenyl butyrate (*p*-NPB), taurocholate, neucuproine, copper (II) chloride, ammonium acetate, iron (III) chloride, acetic acid, hydrochloride, orlistat, simvastatin, pancreatic cholesterol esterase, crude porcine pancreatic lipase, and Tris-HCl (Sigma-Aldrich) have been used. BMG Labtech Spectrostar Nano spectrophotometer used to measure absorbance. pH meter (Hanna Instruments microprocessor), magnetic stirrer (Heidolph MR), analytical weights (Ohaus Pioneer) were also used. Nuclear magnetic resonance (NMR) spectra have been obtained by Bruker Ascend^TM^ 400 MHz/54 mm ULH. Sephadex LH-20 (Sigma-Aldrich) and silica gel (Kiesel gel 60, 0.063-0.2 mm Merck, 7734 and 0.040-0.063 mm Merck, 9385 and LiChroprep RP-18, 25-40 µm, Merck, 9303) for column chromatography (CC) and silica gel 60 F_254_ (Merck, 5554) for thin layer chromatography (TLC) were used. TLC points were determined using a UV lamp and sputtering 1% vanillin/H_2_SO_4_ followed by heating at 110 °C for 1-2 min.

**Plant Material**

*T. orientalis* was collected from Sümer village (Fındıklı, Rize, Turkey) in May 2019 and its authentication was conducted by Prof. Ufuk ÖZGEN, one of the authors. The voucher specimen (No. KATO 15486) was stored at the KATO Herbarium (Karadeniz Technical University, Faculty of Forestry), Trabzon, Turkey.

**Extraction and Fractionation**

The aerial parts of the plant were air-dried in the shade (for approximately one week), ground, and then extracted with methanol (2 L × 3) using a shaker, followed by filtration of the resulting powder (350 g). The methanol extract (TOM) was evaporated to dryness at 40 °C under vacuum conditions. TOM was fractionated by the liquid-liquid partition technique with several solvents ranging from apolar to polar. The TOM (34.5 g) was suspanded in 300 mL of H_2_O:MeOH (9:1) and partitioned with chloroform (300 mL × 2) to get the chloroform subextract (TOC). The aqueous phase was extracted with ethyl acetate (300 mL × 2) to provide the ethyl acetate subextract (TOE). The resultant phases were evaporated with a rotary evaporator, producing TOC (8.9 g), TOE (1.5 g), and the remaining aqueous subextract (TOA, 22.9 g).

**Isolation Procedures**

Following fractionation, TOC (8.5 g) was subjected to chromatography on silica gel column, eluted with *n*-hexane:EtOAc (95:5 to 50:50), resulting in 86 fractions. Fractions 24-33 were amalgamated according to TLC profiles and subsequently submitted to silica gel CC utilizing *n*-hexane:EtOAc with gradient elution (8:2→5:5), resulting in 17 fractions. Fraction 9 yielded compound **1** (27.8 mg).

TOE (1 g) was subjected to CC on Sephadex LH-20. Elution was conducted using methanol. Twenty fractions were gathered. Fraction 12 yielded compound **2** (45.7 mg).

TOA (22.5 g) was subjected to vacuum liquid chromatography on silica gel, with a gradient elution of water:methanol (100:0→0:100). One hundred fractions were gathered. Fractions 12-28 were consolidated and subjected to silica gel CC utilizing CHCl_3_:MeOH:H_2_O (90:10:1, 80:20:2, 70:30:3, 50:50:5) solvent solutions as the eluent. Fractions 63-71 were combined, and compound **3** (10 mg) was further purified. The extraction and isolation processes are illustrated in Figure S1.

Thin Layer Chromatography (TLC) was employed to identify the chemicals in the isolated fractions and to assess their purity (mobile phases: EtOAc:MeOH:H_2_O 7:2:1, *n*-hexane:EtOAc 8:2). The structural elucidation of the compounds was conducted utilizing spectrum techniques, including 1D (^1^H-NMR and ^13^C-NMR) and 2D-NMR (COSY, HMBC, HSQC), validated by comparison with published data.


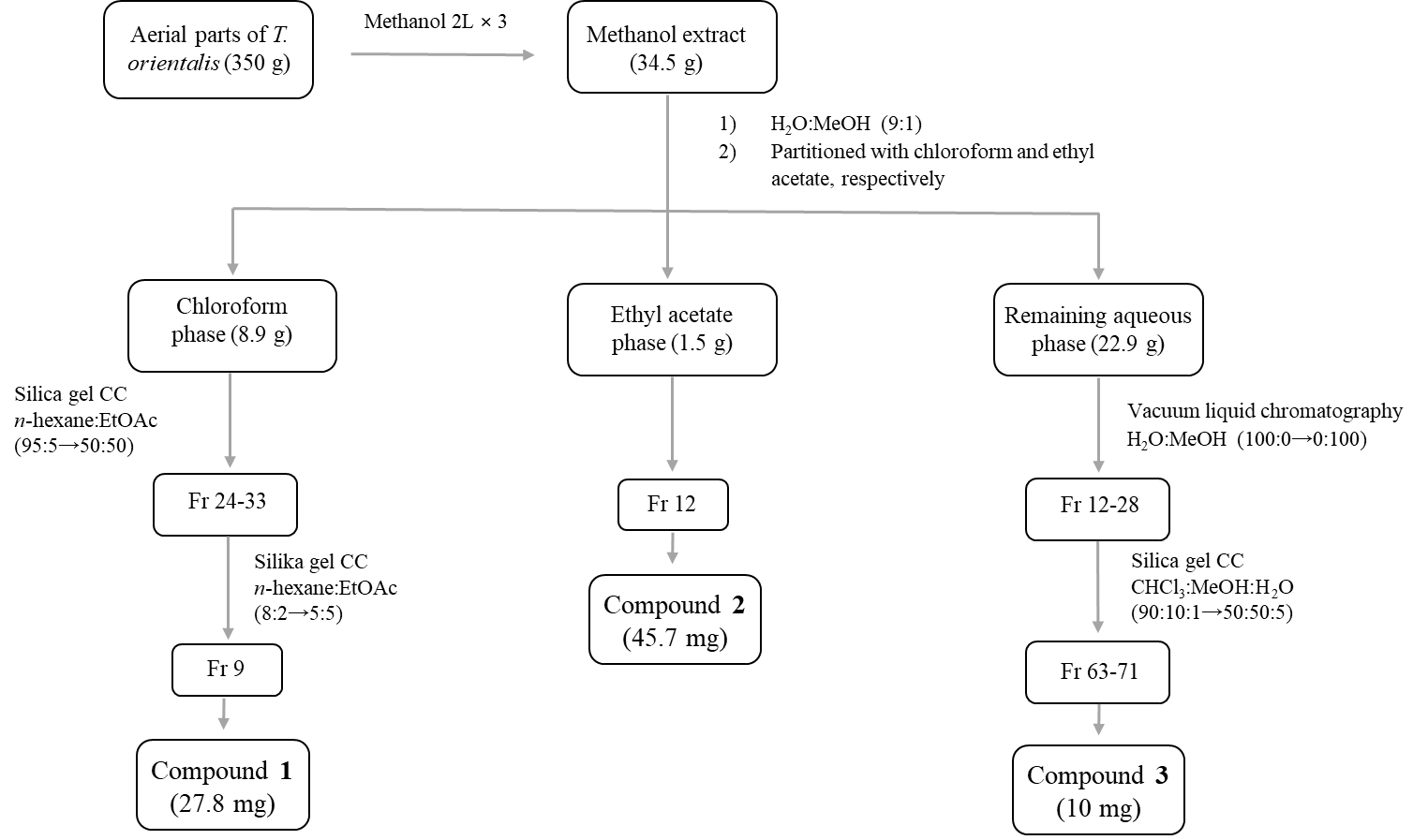


**Figure S1.** The isolation procedure of the aerial parts of *T. orientalis*.

**Compound 1:** C_29_H_50_O (mol. wt. 414); ^1^H-NMR (400 MHz, CDCl_3_) δ: 5.33 (*d*, 1H, H-6, *J*=5.0 Hz), 3.50 (*m*, 1H, H-3), 2.24 (*m*, 2H, H-4), 1.96 (*m*, 2H, H-12), 0.66 (*s*, 3H, H-18); ^13^C NMR (100 MHz, CDCl_3_) δ: 141.0 (C-5), 121.9 (C-6), 72.0 (C-3), 57.0 (C-14), 56.3 (C-17), 50.4 (C-9), 46.1 (C-24), 42.5 (C-4, 13), 40.0 (C-12), 37.5 (C-1), 36.7 (C-10), 36.4 (C-20), 34.2 (C-22), 32.1 (C-7, C-8), 31.9 (C-2), 29.4 (C-25), 28.5 (C-16), 26.3 (C-23), 24.5 (C-15), 23.3 (C-28), 21.3 (C-11), 20.0 (C-27), 19.6 (C-26), 19.6 (C-19), 19.0 (C-21), 12.2 (C-29), 12.1 (C-18). ^1^H-NMR, and ^13^C-NMR data (Figure S2, S3) are in agreement with previously published data for *β*-sitosterol [1,2].

**Compound 2:** C_18_H_16_O_8_ (mol. wt. 360); ^1^H-NMR (400 MHz, CD_3_OD): δ 7.41 (*d*, 1H, H-7, *J*=15.9 Hz), 6.93 (*d*, 1H, H-2, *J=*2.1 Hz), 6.81 (*dd*, 1H, H-6, *J*=8.2 Hz, *J=*2.1 Hz), 6.69-6.65 (*m*, 2H, H-5 and H-2′), 6.58 (*d*, 1H, H-5′, *J*=8.0 Hz), 6.53 (*dd*, 1H, H-6′, *J*=8.1 Hz, *J=*2.0 Hz), 6.17 (*d*, 1H, H-8, *J*=15.9 Hz), 5.00 (*dd*, 1H, H-8′, *J*=9.6 Hz, *J=*3.4 Hz), 3.01 (*dd*, 1H, H-7′a, *J*=14.3 Hz, *J=*3.5 Hz), 2.85 (*dd*, 1H, H-7′b, *J*=14.2 Hz, *J*=9.6 Hz); ^13^C-NMR (100 MHz, CD_3_OD): δ 177.8 (C-9′), 169.2 (C-9), 149.5 (C-4), 146.9 (C-3), 146.8 (C-7), 146.1 (C-3′), 144.9 (C-4′), 131.2 (C-1′), 128.1 (C-1), 123.1 (C-6), 121.9 (C-6′), 117.7 (C-2′), 116.6 (C-5), 116.3 (C-5′), 115.6 (C-2), 115.3 (C-8), 77.6 (C-8′), 38.8 (C-7′). ^1^H-NMR and ^13^C-NMR data (Figure S4, S5) are consistent with literature data for Rosmarinic acid [2,3].

**Compound 3 (a mixture):** ^1^H-NMR and ^13^C-NMR data of **rosmarinic acid** in the mixture are in agreement with the data above. **Danshensu:** C_9_H_10_O_5_ (mol. wt. 198); ^1^H-NMR (400 MHz, CD_3_OD): δ 6.71-6.49 (*m*, 7H, H-2, H-5, H-6 overlapped H-R5, H-R2′, H-R5′, H-R6′), 3.99 (*dd*, 1H, H-2′, *J*=8.2 Hz, *J*=3.5 Hz), 2.92-2.84 (*m*, 2H, H-3′a overlapped R7′b), 2.57 (*dd*, 1H, H-3′b, *J*=13.9 Hz, *J*=8.2 Hz); ^13^C-NMR (100 MHz, CD_3_OD): δ 180.7 (C-1′), 146.7 (C-3), 146.0 (C-4), 131.9 (C-1), 122.1 (C-6), 117.6 (C-2), 116.2 (C-5), 74.8 (C-2′), 41.9 (C-3′). ^1^H-NMR, ^13^C-NMR, COSY, HSQC, and HMBC data (Figure S6-S12) are agreement with the previously data in the literature for Danshensu [4,5].


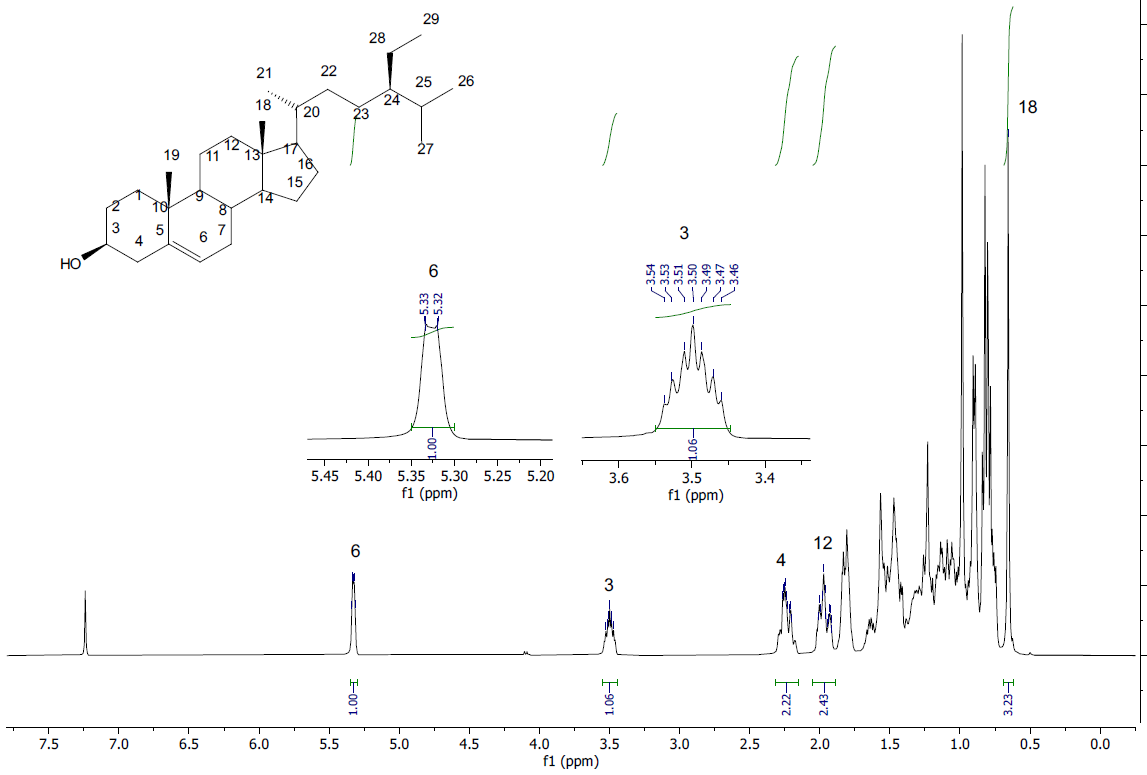


**Figure S2.** ^1^H-NMR of *β*-sitosterol (CD_3_OD, 400 MHz)


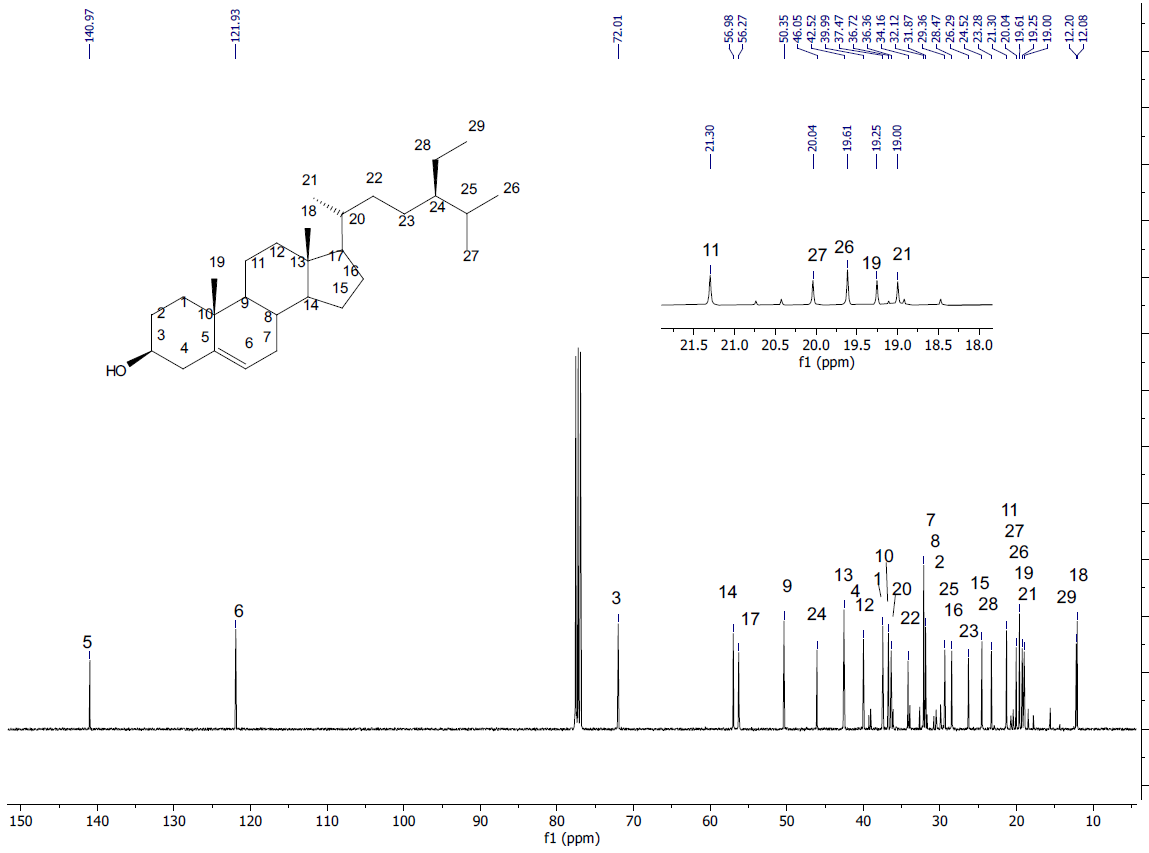


**Figure S3.** ^13^C-NMR of *β*-sitosterol (CD_3_OD, 100 MHz)


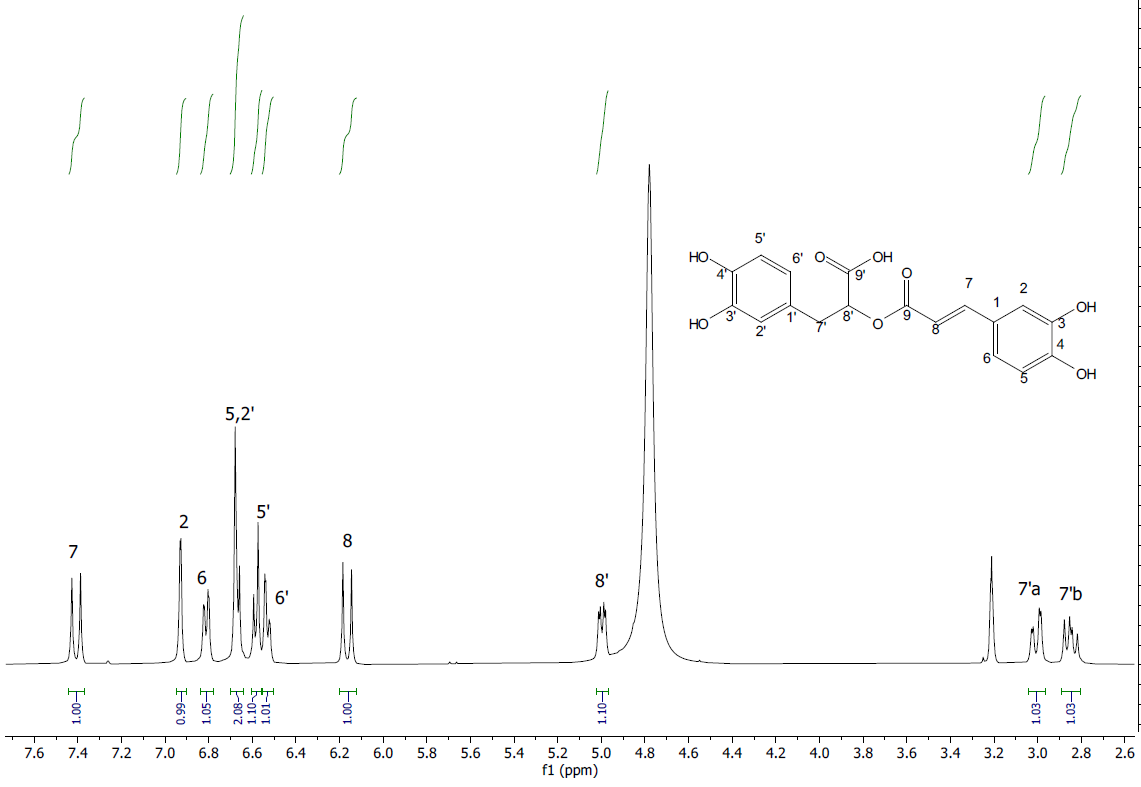


**Figure S4.** ^1^H-NMR of Rosmarinic acid (CD_3_OD, 400 MHz)


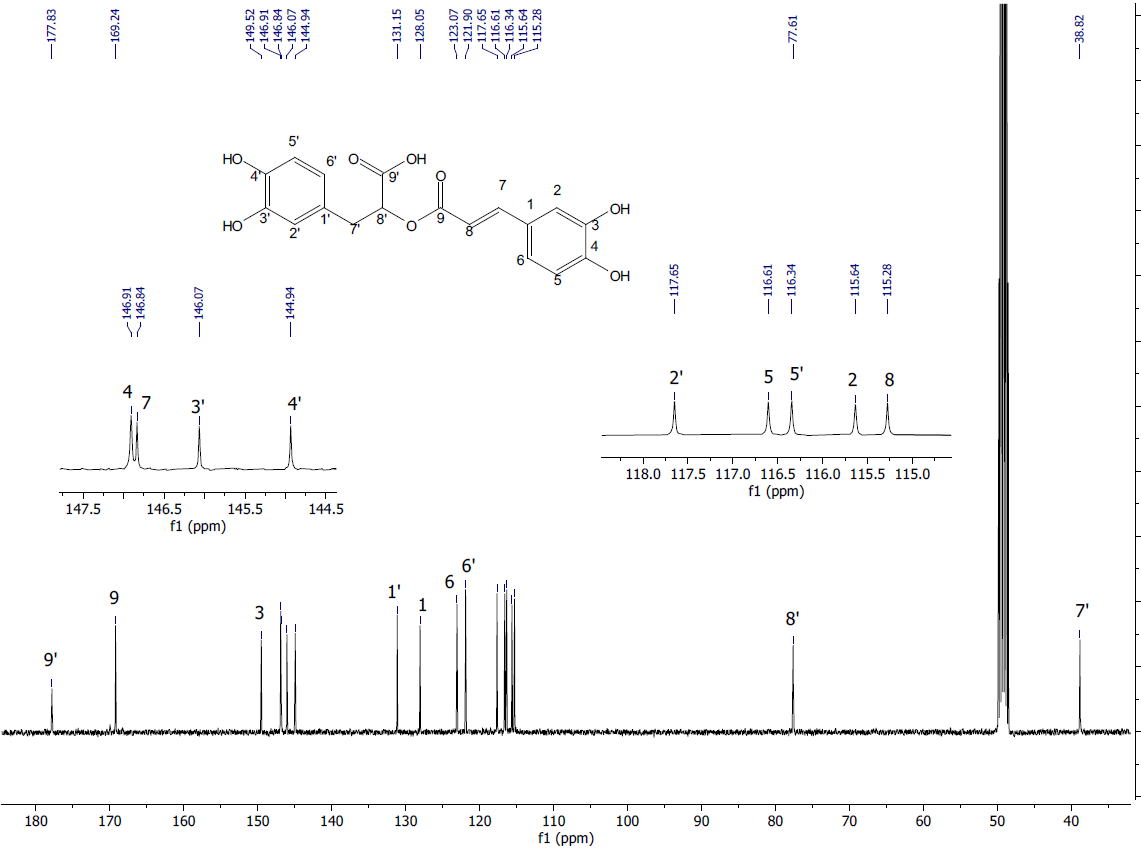


**Figure S5.** ^13^C-NMR of Rosmarinic acid (CD_3_OD, 100 MHz)


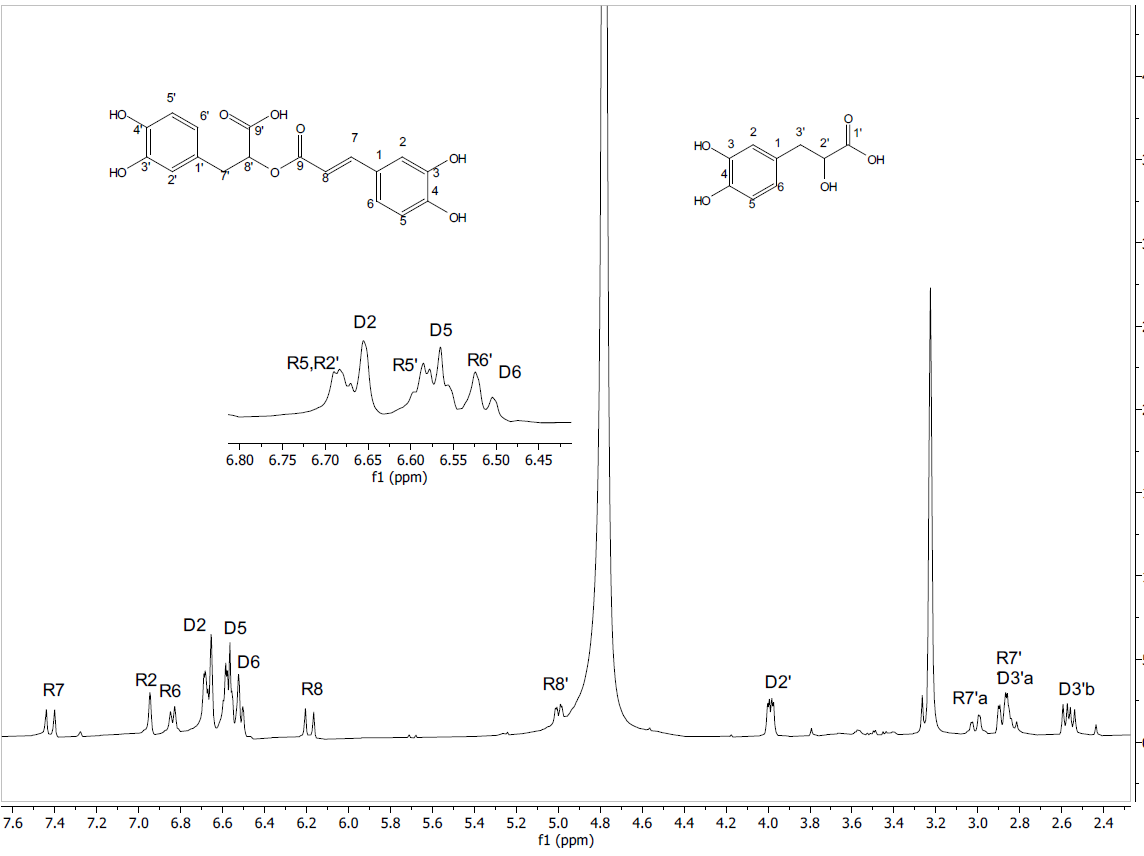


**Figure S6.** ^1^H-NMR of the mixture of Rosmarinic acid and Danshensu (CD_3_OD, 400 MHz)


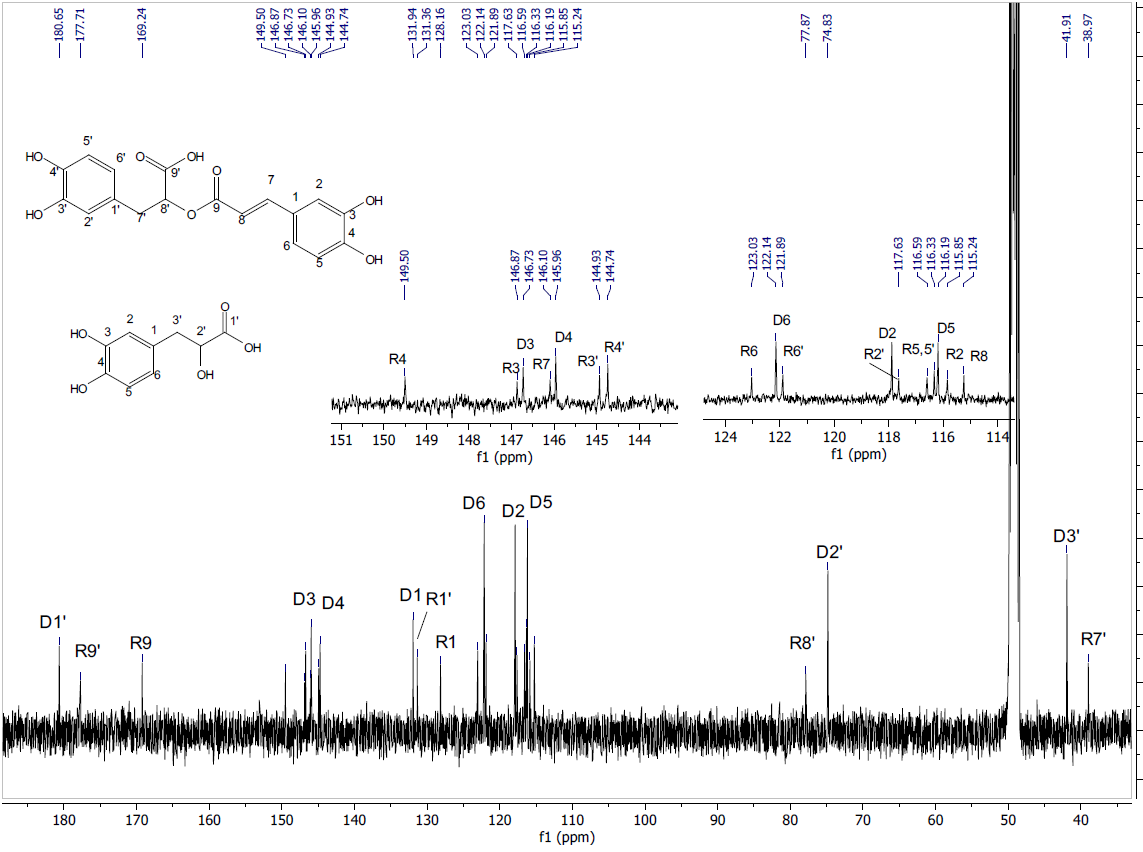


**Figure S7.** ^13^C-NMR of the mixture of Rosmarinic acid and Danshensu (CD_3_OD, 100 MHz)

**
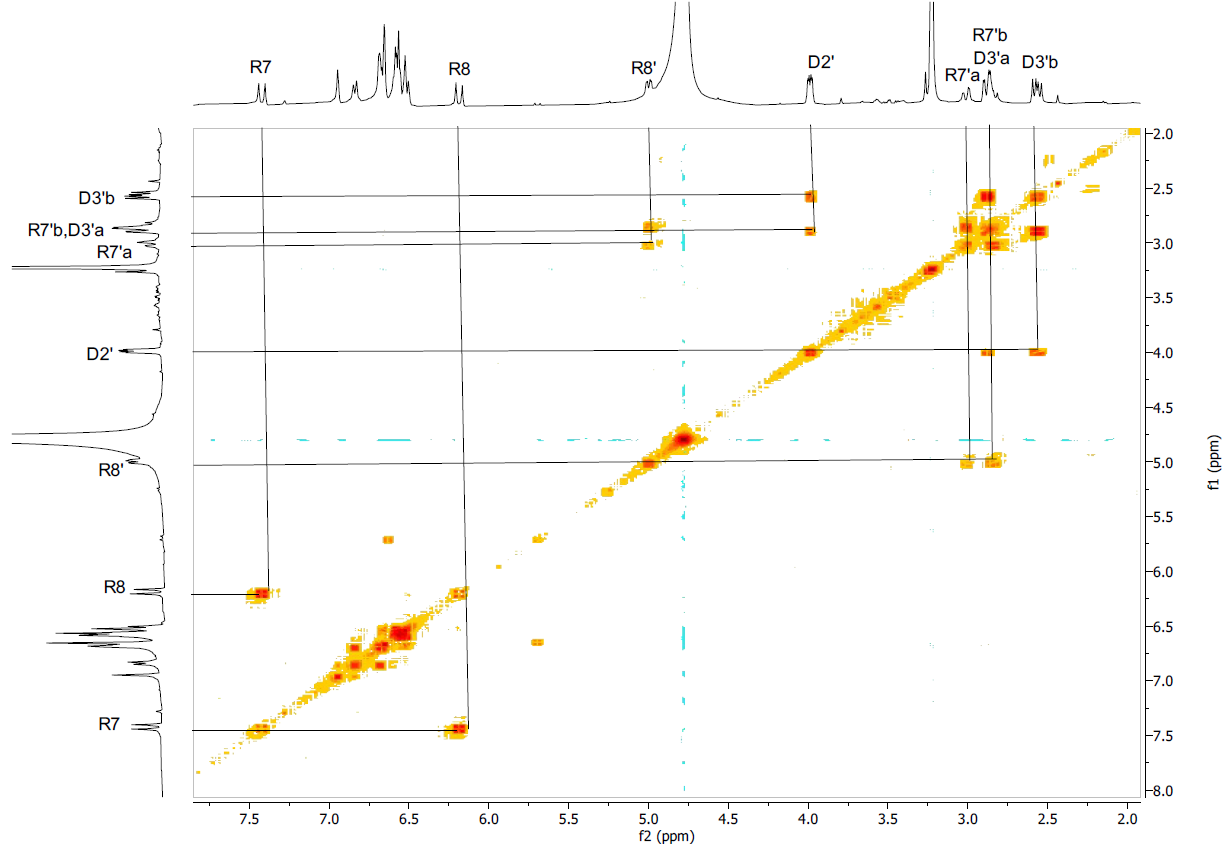
**

**Figure S8.** COSY spectrum of the mixture of Rosmarinic acid and Danshensu


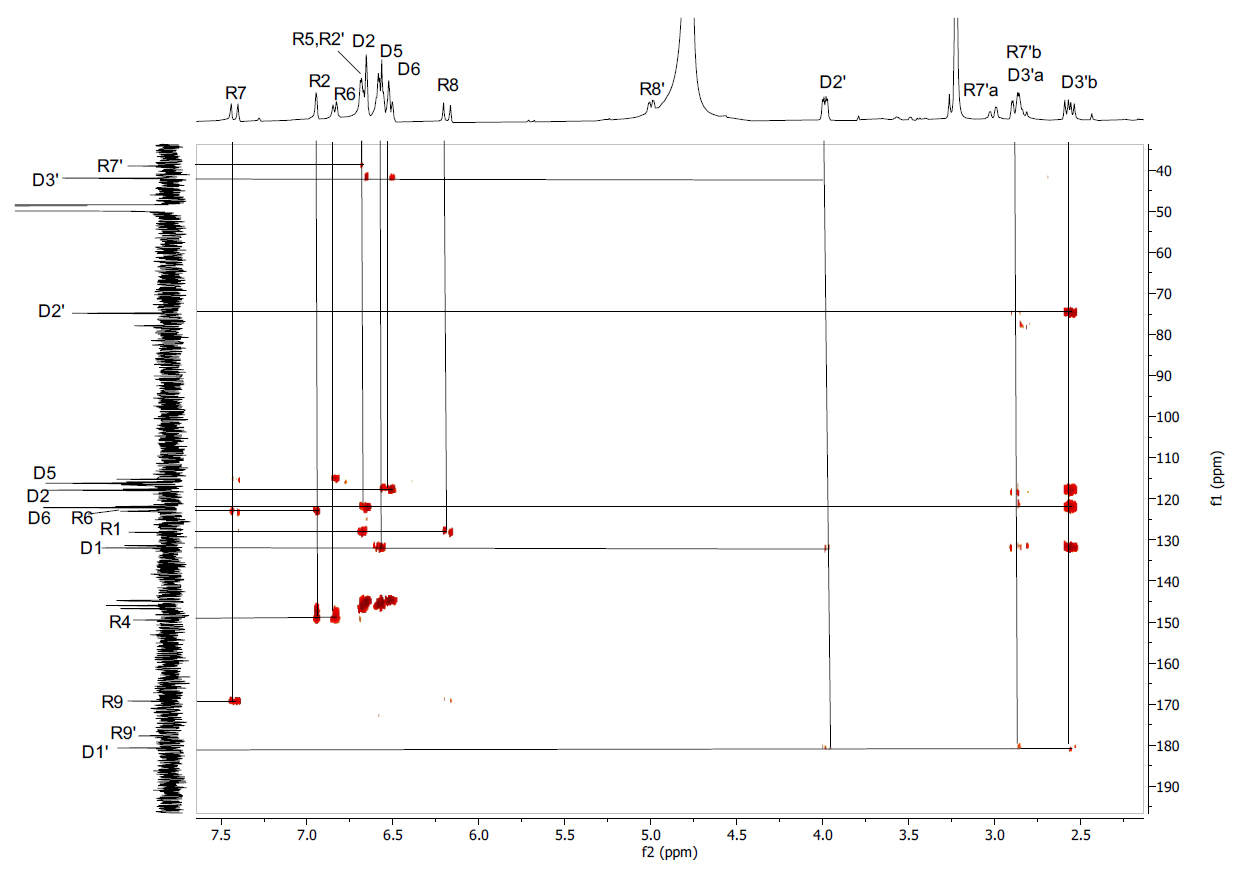


**Figure S9.** HMBC spectrum of the mixture of Rosmarinic acid and Danshensu


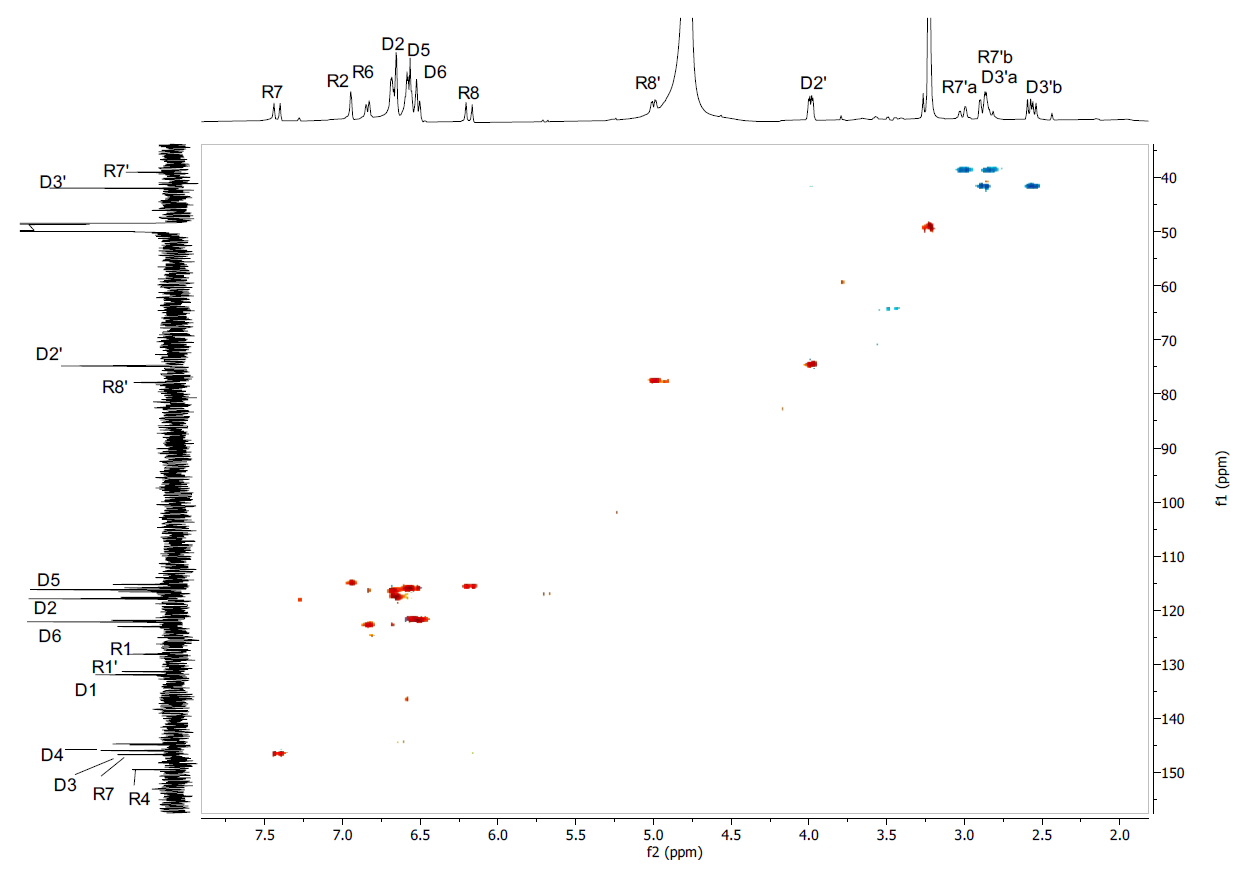


**Figure S10.** HSQC spectrum-I of the mixture of Rosmarinic acid and Danshensu


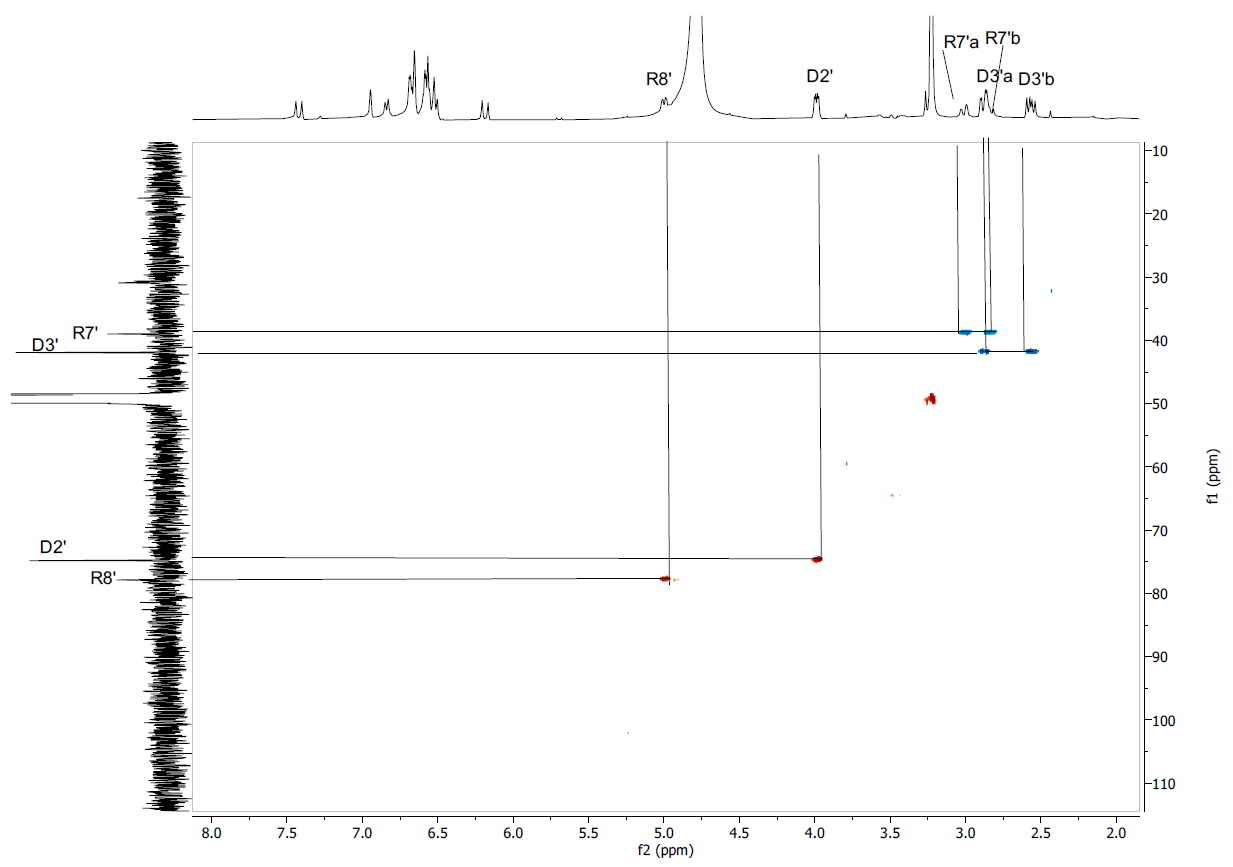


**Figure S11.** HSQC spectrum-II of the mixture of Rosmarinic acid and Danshensu


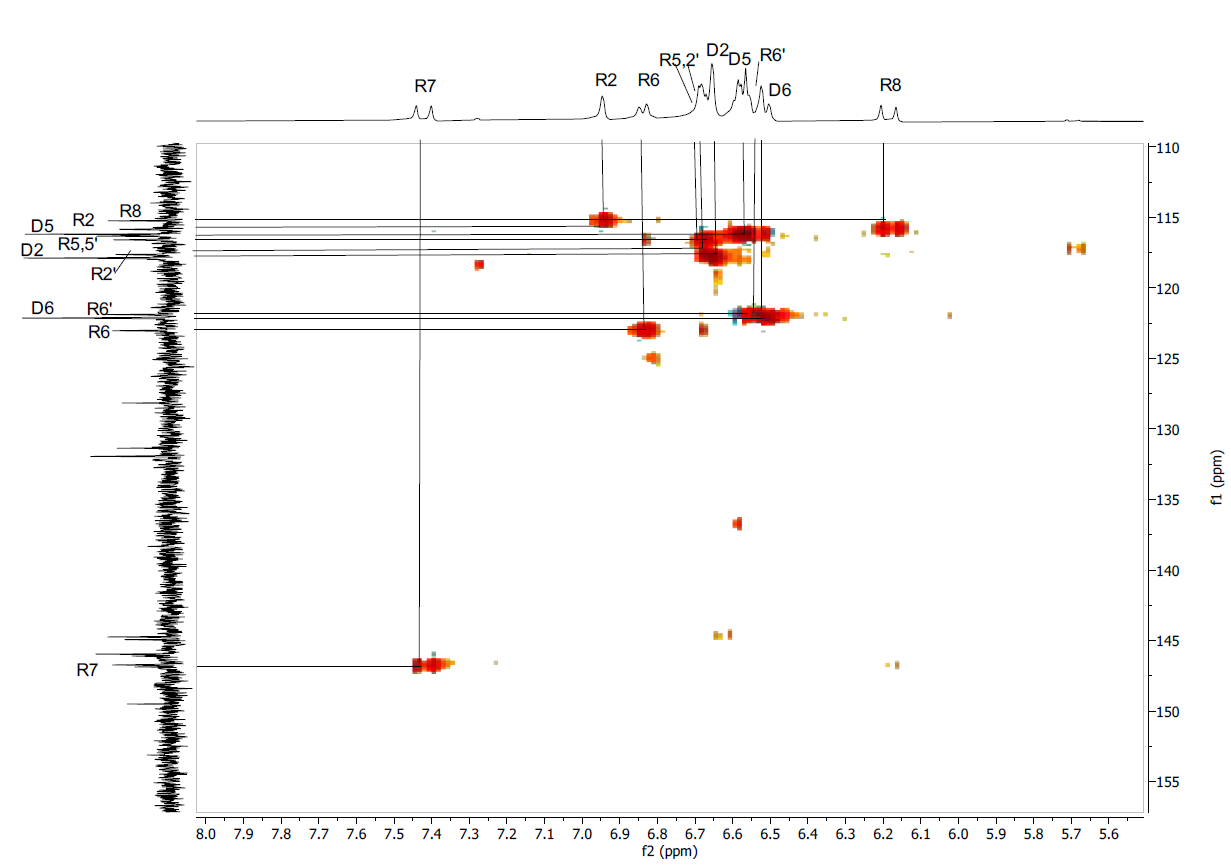


**Figure S12.** HSQC spectrum-III of the mixture of Rosmarinic acid and Danshensu

**Pancreatic Lipase (PL) Inhibition**

PL inhibition was assessed utilizing *p*-nitrophenyl butyrate as a substrate [6]. The extracts were diluted using a buffer solution (0.1 M Tris-HCl, pH 8.0) to achieve a final concentration range of 12.5-400 µg/mL. Orlistat was utilized as a standard at several concentrations (6.25-100 µg/mL). An enzyme solution (EC 3.1.1.3, 200 units/mL) and a substrate solution (10 mM) were formulated in a buffer solution.

The experimental procedure was structured in microplate wells A (90 µL enzyme solution, 5 µL substrate solution, 5 µL buffer solution), B (90 µL enzyme solution, 10 µL buffer solution), C (90 µL enzyme solution, 5 µL sample solution, 5 µL substrate solution), and D (90 µL enzyme solution, 5 µL sample solution, 5 µL buffer solution). Microplates were incubated at 37 °C for 15 minutes before to and following the addition of substrates. The absorbance values were measured at 405 nm, and the percentage of pancreatic lipase enzyme inhibition was calculated. All concentrations were conducted in triplicate.

% Pancreatic lipase inhibition = [(A-B)-(C-D)/(A-B)] × 100

The IC_50_ values of the samples on lipase were derived using the graph equation established by plotting the observed percentage of enzyme inhibition against the logarithm of the concentrations (ordinate and abscissa).

**Cholesterol Esterase (CE) Inhibition**

CE inhibition was determined spectrophotometrically using a modified approach [7-9]. Samples (5-80 µg/mL), CE enzyme (from porcine pancreas, EC 3.1.1.13, 35 U/mg) at a concentration of 1 µg/mL, and taurocholic acid (12 mM) were solubilized in 100 mM phosphate buffer (0.1 M NaCl, pH 7), while a 10 mM *p*-NPB (substrate) solution was formulated in the identical buffer. Simvastatin was utilized as a positive control at varying doses (5-160 µg/mL).

Following the addition of 10 µL of sample solution, 100 µL of taurocholic acid solution, 20 µL of buffer, and 10 µL of substrate solution to the microplate wells, the mixture was incubated at 37 °C for 10 minutes. Upon completion of incubation, 10 µL of enzyme solution was introduced to each well and incubated at 37 °C for 25-30 minutes. The samples' absorbance was measured at 405 nm with a spectrophotometer. Each sample was executed in three parallel trials. The experimental findings were averaged, and the percentage inhibition values were computed using the formula shown below. The IC_50_ values of the samples were determined using a graph constructed using the computed percentage inhibition data and the logarithmic concentrations of the samples.

% Cholesterol esterase inhibition = [(A_control_-A_sample_)/A_control_] × 100

**Determination of Antioxidant Capacity**

*The Ferric Reducing Antioxidant Power (FRAP) Assay*

The FRAP assay principle involves quantifying the iron reduction capability of the samples [10]. The Fe(II)-TPTZ complex, generated by the reduction of the TPTZ complex in the presence of antioxidants, has a blue colour and is measured at 595 nm. Fifty microliters of sample solution, standard blank, and 1.5 mL of FRAP reagent were pipetted from tests and samples (10 mg/mL) into tubes. Absorbance measurements were recorded at 595 nm after 20 minutes at room temperature. The FRAP values of the samples were ascertained from the graph depicting concentration against absorbance, utilizing Trolox solutions (standard) at varying concentrations (62.5-1000 µM). FRAP values were reported as μM Trolox equivalents per gram of material.

*Cupric-Reducing Antioxidant Capacity (CUPRAC) Assay*

CUPRAC assay was performed according to the modified protocol [11-13]. Methanolic solutions of Trolox, utilized as a reference, were produced at varying concentrations (1000-62.5 μM). Subsequently, 1 mL of NH_4_CH_3_COO and 1 mL of CuCl_2_.2H_2_O and 1 of mL neocuproine (Nc) solutions were combined, followed by the addition of 0.5 mL of the sample solution (10 mg/mL) and 0.6 mL of distilled water (1.1-x) water into the mixture. Following the mixing of the tubes and incubation in darkness at ambient temperature for 30 minutes, the absorbance of the samples was measured at 450 nm. The antioxidant capacity of the samples was quantified as TEAC (μM) by comparison with Trolox, the reference chemical. The calibration curves for FRAP and CUPRAC assays are presented in Figure S13.


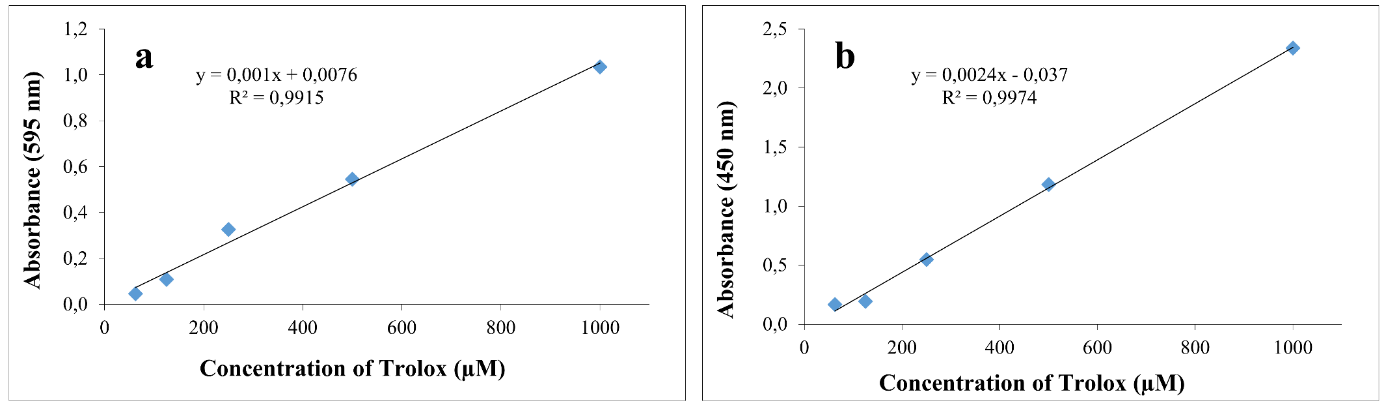


**Figure S13.** Calibration curves for FRAP (**a**) and CUPRAC (**b**) assays.

**Statistical analysis**

All samples were analyzed in triplicate, and the experimental data are reported as mean ± standard deviation (SD). IC_50_ values were determined with Microsoft Excel. Statistical differences were assessed using one-way ANOVA, followed by Tukey's multiple comparisons test, utilizing GraphPad Prism 8.0.2 (GraphPad Prism Software, San Diego, CA, USA). Statistical significance was established at *p*<0.05.

**Additional References**

1. Aliba MO, Ndukwe IG, Ibrahim H (2018) Isolation and characterization of *β*-sitosterol from methanol extracts of the stem bark of large- leaved rock fig (*Ficus abutilifolia* Miq). JASEM 22:1639. <https://doi.org/10.4314/jasem.v22i10.19>
2. Badem M, Sener SO, Kanbolat S, Korkmaz N, Yildirmiş S, Ozgen U, Aliyazicioglu R, Salva, E, Kaban K, Kandemir A, Calıs I (2021) Evaluation of biological activities of *Barbarea integrifolia* and isolation of a new glucosinolate derivated compound. Z Naturforsch C J Biosci 76(9-10):375-382. <https://doi.org/10.1515/znc-2020-0305>
3. Sezen Karaoglan E, Ozgen U, Kazaz C (2017) Phytochemical studies on *Origanum rotundifolium*. Bangladesh J Pharmacol 12(4):470-471. <https://doi.org/10.3329/bjp.v12i4.34249>
4. Sidoryk K, Filip K, Cmoch P, Laszcz M, Cybulski M (2018) Efficient synthesis and physicochemical characterization of natural danshensu, its S isomer and intermediates thereof. J Mol Struct 1153:135-148. <https://doi.org/10.1016/j.molstruc.2017.09.118>
5. Koz AN, Subaş T, Badem M, Kanbolat Ş, Özgen U, Şener SÖ, Demir A, Çalış İ (2024) Isolation of the major compounds and determination of biological activities in the underground parts of *Trachystemon orientalis* D.Don. Turk J Pharm Sci 21(6):544-550. <https://doi.org/10.4274/tjps.galenos.2024.60980>
6. Bustanji Y, Al-Masri IM, Mohammad M, Hudaib M, Tawaha K, Tarazi H, Alkhatib HS (2011). Pancreatic lipase inhibition activity of trilactone terpenes of *Ginkgo biloba*. J Enzyme Inhib Med Chem 26(4):453-459. <https://doi.org/10.3109/14756366.2010.525509>
7. Pietsch M, Gütschow M (2005) Synthesis of tricyclic 1,3-oxazin-4-ones and kinetic analysis of cholesterol esterase and acetylcholinesterase inhibition. J Med Chem 48(26):8270-8288. <https://doi.org/10.1021/jm0508639>
8. Ngamukote S, Mäkynen K, Thilawech T, Adisakwattana S (2011) Cholesterol-lowering activity of the major polyphenols in grape seed. Molecules 16(6):5054-5061. <https://doi.org/10.3390/molecules16065054>
9. Zor M, Pekacar S, Deliorman Orhan D (2022) *Nasturtium officinale*’nin fitokimyasal içeriği, antioksidan ve enzim inhibitör etkileri. Ankara Ecz Fak Derg 46(1):114-128. <https://doi.org/10.33483/jfpau.1018949>
10. Benzie IF, Strain JJ (1999) Ferric reducing/antioxidant power assay: direct measure of total antioxidant activity of biological fluids and modified version for simultaneous measurement of total antioxidant power and ascorbic acid concentration. Meth Enzymol 299:15-27. <https://doi.org/10.1016/s0076-6879(99)99005-5>
11. Apak R, Güçlü K, Demirata B, Ozyürek M, Celik SE, Bektaşoğlu B, Berker KI, Ozyurt D (2007) Comparative evaluation of various total antioxidant capacity assays applied to phenolic compounds with the CUPRAC assay. Molecules 12(7):1496-547. <https://doi.org/10.3390/12071496>
12. Bener M, Şen FB, Önem AN, Bekdeşer B, Çelik SE, Lalikoglu M, Aşçı YS, Capanoglu E, Apak, R (2022) Microwave-assisted extraction of antioxidant compounds from by-products of Turkish hazelnut (*Corylus avellana* L.) using natural deep eutectic solvents: Modeling, optimization and phenolic characterization. Food Chem 385:132633. <https://doi.org/10.1016/j.foodchem.2022.132633>
13. Erdoğan U, Oztürk TH, Onder S, Tonguç M (2024) Eco-friendly extraction of six different rosemary (*Rosmarinus officinalis* L.) genotypes-using natural deep eutectic solvents: Optimization and modeling via response surface methodology (RSM). J Mol Liq 407:125167. <https://doi.org/10.1016/j.molliq.2024.125167>
